# Association between lipodystrophy and length of exposure to ARTs in adult HIV-1 infected patients in Montreal

**DOI:** 10.1186/s12879-019-4446-9

**Published:** 2019-09-18

**Authors:** Ahmad Alikhani, Helene Morin, Stephanie Matte, Pouriya Alikhani, Cécile Tremblay, Madeleine Durand

**Affiliations:** 1 Department of infectious diseases &antimicrobial resistance research center, Mazandaran University of Medical Sciences, Sari, Iran; 2 CHUM-CRCHUM, Tour Saint-Antoine - 3e étage, local SO3-288, 850, rue Saint-Denis, Montréal, Québec H2X 0A9 Canada; 3 McGill Centre for bioinformatics, McGill University, Montreal, Canada

**Keywords:** HIV, Antiretroviral therapy, Lipodystrophy, Risk factors, Dyslipidemia

## Abstract

**Background:**

The aim of this study was to establish the prevalence of lipodystrophy and its association to cumulative exposure to antiretroviral drugs.

**Method:**

We conducted a cross sectional study in all HIV- infected patients attending the HIV clinic in the Centre hospitalier universitaire de Montréal (CHUM) with DEXA scan. Lipodystrophy was defined as a trunk/limb fat ratio ≥ 1.5. Association between cumulative exposure to antiretroviral (measured in years of use) with trunk/limb fat ratio (coded as a continuous variable) was assessed using univariate and multivariate linear regression for each antiretroviral drug with at least 40 exposed patients.

**Results:**

One hundred sixty-six patients were included. Seventy-five percent were male, median age was 56 years, 67% were Caucasian. Overall, prevalence of lipodystrophy was 47%, with a mean trunk/limb fat ratio of 1.87, SD = 1.03, min = 0.6 and max = 5.87. Each 10-year increase in age and HIV infection duration was associated with an average increase of 0.24 and 0.34 for the trunk/limb fat ratio respectively. (*p* = 0.003, *p* = 0.002, respectively) Patients classified as lipodystrophic were more likely to be diabetic (50 vs. 28%, *p* = 0.07) and to have dyslipidemia (47 vs. 19%, *p* = 0.01). According to viral load at DEXA test, each one log increase was associated with less probability (0.7) of lipodystrophy. (p = 0.01) Among ARV drugs tested, there was an association between years of use of d4T, ritonavir and raltegravir and higher trunk/limb fat ratio (indicating more lipodystrophy) (*p* < 0.05).

**Conclusion:**

Lipodystrophy is very common in HIV infected patients and is correlated with duration of some new antiretroviral drugs.

## Background

Combination antiretroviral medications (ARTs) improved the survival and quality of life of HIV infected patients, but they have many side effects. Lipodystrophy and metabolic abnormalities, among other adverse reactions may lead to cardiovascular problems [1–5].

About 20,000 people are living with HIV in Quebec and are taking combination antiretroviral therapies that maybe lead to LD [6]. Lipodystrophy [lipoatrophy (LA) and lipohypertrophy (LH)] has been most associated with zidovudine and stavudine (LA) and ritonavir (LH), [7, 8] which usually occurs months to years after ARTs introduction and prevalence rates vary from 18 to 83% [8–10]. It is associated with hyperglycemia [11–13], hyperlipidemia [11], HIV infection and its duration [8, 14], level of immunity [15, 16], female gender [15], increasing age [9, 16] and type and duration of ARTs [16, 17].

LD causes body changes which are usually associated with a decrease in self-esteem and leads to decreasing of adherence to ART and is associated to cardiovascular problems [1] This syndrome can also impact on quality of life, causes mental health disorders [10] and ultimately lead to treatment failure [1, 10]. Dual energy X-ray absorptiometry (DEXA) scan is a reliable method for definition of this syndrome [9].

We aimed to establish the frequency of LD in the population followed at our HIV clinic and identify risk factors associated with deterioration of fat distribution.

## Methods

### Study design

This is a cross sectional study nested within the cohort of all HIV positive individuals followed at our clinic from 2011 to 2015.

#### Source and study population

The HIV clinic of the CHUM has offered care to approximately 3500 people living with HIV from its start in 1985. Antiretroviral drugs were available to its patients since the approval of zidovudine in Canada in 1987, and access to combination therapies was concomitant to their approval in Canada. Starting in 2011 and until 2015 (end of data collection for this study), DEXA scans have been available to patients followed in the HIV clinic for assessment of bone health and lipodystrophy. DEXA scans were routinely offered to patients over 40 years of age or with risk factors for osteoporosis. It is estimated that during the study period, approximately 700 patients had visits at the clinic, of which 166 underwent DEXA scans for clinical reasons. All patients who underwent DEXA scans were eligible to participate in this study.

#### Study procedures

DEXA scans were all performed using the same device, and measured total body fat and fat distribution. Fat distribution was given as the ratio of fat contained in trunk to that contained in limbs (fat mass ratio [FMR]). All patients consented to contribute data to the database, which was approved by the CHUM research ethic board.

#### Data collection

The following clinical data were obtained in the clinic database: age, gender, race, duration of HIV infection, presence and duration of diabetes mellitus, presence of hypercholesterolemia and hypertriglyceridemia, and duration of exposure to all antiretroviral agents. The following laboratory values were also extracted: fasting lipid profiles (triglyceride, total cholesterol, LDL and HDL), fasting blood sugar, CD4 and CD8 lymphocyte counts (nadir and values closest to DEXA scan), plasma HIV RNA before antiretroviral initiation and closest to DEXA scan, and years spent with undetectable HIV RNA level. Closest to DEXA scan was within 3 months and for all the values within one year.

#### Outcome definitions

The outcome of interest in all analyses was LD. To contrast population characteristics in individuals with and without established LD, it was defined in a dichotomous way using the published FMR cut-off point of 1.5. Values above or equal to 1.5 for FMR were considered as diagnostic of LD [18]. However, to analyze the impact of individual risk factors on LD, the FMR was kept continuous, with higher values of FMR indicating more LD, and lower values of FMR indicating less LD.

#### Covariates selection

Covariates were separated based on clinical knowledge and direct acyclic graphs as covariates potentially driving LD (explanatory variables): age, sex, immuno-virologic factors (years of HIV infection, CD4 and CD8 lymphocyte count, plasma HIV RNA, and exposure to ARVs). Variables that captured markers of metabolic dysfunction potentially resulting from LD (co-morbidity variables) such as blood lipids and blood sugar were assessed separately.

#### Statistical analysis

Appropriate summary statistics were used to describe the characteristics of patients included into the study, stratified by presence or absence of LD. Characteristics of patients with (defined as FMR > =1.5) or without LD (defined as FMR < 1.5) were compared using t-tests (for continuous variables), chi-square tests (for categorical variables) or Fisher’s exact tests (for categorical variable with small cell counts), as appropriate.

Association between explanatory variables and FMR (modeled as continuous) were assessed using uni and multivariable linear regression. Exposure to antiretrovirals was captured as a continuous measure of years of medication exposure. Antiretroviral medications were analyzed only if at least 40 patients from our sample had been exposed to any given molecule. For the multivariable model, explanatory variables were entered in the multivariable model if they showed an association (*p* ≤ 0.10) with FMR in the univariable model, and kept into the final model if they were independently associated with FMR or if they modified the point estimate for other predictors by more than 10%.

Association between FMR and co-morbidity variables (fasting blood sugar, total cholesterol, LDL, HDL) were assessed in a separate linear regression model, as those anomalies are more likely secondary to LD rather than as risk factors for LD. Analysis was performed using Stata 11. All statistical tests were two-sided, with a p level of 0.05. No adjustments for multiple testing were done.

#### Post hoc power calculations

The sample size was not determined in advance, as our study was conducted on a convenience sample of all patients with available DEXA scans from our clinic. In post hoc power analysis, we determined that, with alpha set at 0.05 and given our sample size of 166, we had 80% power to detect an odds ratio of lipodystrophy of 1.5 associated to a drug with at least 40 exposed patients. This increased to 95% power for drugs with 80 exposed patients. To detect odds ratios of 1.3, however, power ranged from 36% for drugs with 40 exposed patients to 56% for drugs with 80 exposed patients. Those calculations do not take into account adjustments for multiple testing, and are for crude associations only. Power calculations were done with online calculator *Power and sample size* [19].

## Results

One-hundred and sixty-six patients were included. Seventy-five percent were male, median age was 56 years old (interquartile range [IQR] 50–64), and 67% were Caucasian. Overall, prevalence of lipodystrophy was 47%, with a mean trunk/limb fat ratio of 1.87, SD = 1.03, min = 0.6 and max = 5.87. All were exposed to antiretroviral drugs. Among those 133(79%) were taking ritonavir, 118(71%) zidovudine, 114(69%) tenofovir, 81(49%) lopinavir and 70(42%) atazanavir. One hundred seventeen cases (70.4%) had viral load of less than 50 copies/ml at the time of DXA scan.

### Dichotomous LD definition

Baseline characteristics of patients, stratified by LD status, are presented in Table 1. Patients with LD were older (57.9 vs 54.7, *p* = 0.03.) They were also more likely to be diabetic (50 vs. 28%, Fisher’s exact *p* = 0.07) and to have dyslipidemia (47 vs. 19%, *p* = 0.01) and were more likely to use statins, fibrate and oral antidiabetic drugs. Patients with LD were also more likely to have been exposed to zidovudine, stavudine, abacavir, etravirine, ritonavir, raltegravir and enfuvirtide, while exposure to other ARVs was not different in the two groups.

**Table 1:** Characteristics of study population, stratified by trunk/limb fat ratio less than or equal to 1.5 (no lipodystrophy) or greater than 1.5 (lipodystrophy)

Demographic factors	With lipodystrophy (*N* = 78)	Without lipodystrophy (*N* = 88)	*P* value
Age (Mean, SD)	57.9 (7.3)	54.7 (11.0)	0.033
Male sex (n, %)	73 (93.6)	51 (58.0)	< 0.001
Caucasian ethnicity (n, %)	60 (76.9%)	51 (57.5%)	0.010
Duration of HIV infection (mean, SD)	13.8 (6.65)	12.1 (8.04)	0.14
Concurrent medications (proportion exposed)			
Statins	23 (29.5)	11 (12.5)	0.007
Fibrates	22 (28.2)	8 (9.1)	0.001
Oral anti diabetes	16 (20.5)	5 (5.68)	0.004
Antiretrovirals (proportion exposed)			
Zidovudine	62 (79.5)	56 (63.6)	0.02
Stavudine	36(46.2)	26 (29.6)	0.03
Abacavir	37 (47.4)	29 (32.9)	0.06
Tenofovir	56 (71.8)	58 (65.9)	0.41
Etravirine	12 (15.4)	0 (0.0)	< 0.001
Atazanavir/r	35 (44.9)	35 (39.8)	0.51
Lopinavir/r	42 (53.8)	39 (44.3)	0.22
Darunavir/r	26 (33.3)	19 (21.6)	0.089
Tipranavir	5 (6.4)	1 (1.1)	0.10
Fosamprenavir	2 (2.6)	2 (2.3)	1.0
Nelfinavir	23 (29.5)	16 (18.2)	0.086
Ritonavir	31 (39.7)	21 (23.9)	0.028
Raltegravir	35 (44.9)	23 (26.1)	0.012
Elvitegravir	3 (3.9)	1 (1.1)	0.34
Dolutegravir	2 (2.3)	3 (3.9)	0.67
Maraviroc	10 (12.8)	5 (5.7)	0.11
Enfuvirtide	5 (6.41)	0 (0.0)	0.016

*HIV* human immunodeficiency virus, /*r* ritonavir

### Continuous FMR analysis – explanatory variables

Table 2 shows the results of uni and multivariable linear regression models for the association between explanatory variables and FMR as a continuous variable. The variables that were associated with increased in FMR were age (increase in FMR of 0.24 [95%CI 0.08 to 0.41], and duration of HIV infection (increase in FMR of 0.34 [95%CI 0.13 to 0.55] per 10 years of HIV duration. Factors associated with a decrease in FMR were female sex (women’s mean FMR was lower by 0.78 [95%CI 1.13 to 0.43] than men) and higher viral loads. CD4 levels at the time of DEXA scan and nadir CD4 levels showed no association with FMR. Association between years of cumulative exposure and FMR was tested for 9 ARVs drugs with at least 40 exposed patients. Of those, stavudine, ritonavir, and raltegravir were associated with increases in FMR.

**Table 2:** Uni and multivariable linear regression analysis of patient’s characteristics, years of exposure to antiretrovirals and trunk/limb fat mass ratio in 166 pts. with DEXA scan results

Risk factors	Univariable^a^ regression coefficient [95% CI]	*p*-value	Multivariable^b^ regression coefficient [95% CI]	p- Value
Characteristics of individual and HIV infection				
Age (per 10 years increase of age)	0.24 [0.08;0.41]	0.003	–	–
Sex (female compared to male)	−0.78 [−1.13; − 0.43]	< 0.001	−0.83 [−1.15; − 0.51]	< 0.001
Years since HIV infection (per 10 years of infection duration)	0.34 [0.13;0.55]	0.002	–	–
Nadir CD4 (per 100 cells)	0.02 [− 0.04;0.08]	0.46	–	–
Immune parameters at time of dexa scan				
CD4 at DEXA (per 100 cells)	0.01 [−0.05;0.08]	0.70	–	–
logViral load at DEXA test (per 1 log increase)	−0.7 [− 0.13; − 0.01]	0.015	−0.05 [− 0.11;0.00]	0.058
Exposure to antiretrovirals (measured in years of exposure, coefficients for 10 years increase in cumulative use)				
Zidovudine	0.26 [−0.05;0.57]	0.10	0.34 [0.06;0.62]	0.018
Stavudine	1.0 [0.41;1.59]	0.001	0.92 [0.38;1.47]	0.001
Abacavir	0.00 [−0.41;0.41]	1.0	–	–
Tenofovir	−0.05 [− 0.44;0.33]	0.78	–	–
Lopinavir/r	− 0.20 [− 0.61;0.20]	0.33	–	–
Atazanavir/ r	0.11 [− 0.30;0.53]	0.59	–	–
Draunavir/r	0.31 [− 0.27;0.90]	0.30	–	–
Ritonavir	0.64 [0.12;1.17]	0.017	–	–
Raltegravir	0.71 [0.19;1.22]	0.008	0.57 [0.10;1.05]	0.019

DEXA Dual Energy Xray Absorbimetry; HIV human immunodeficiency virus; /r ritonavir

^a^Univariable models are models with only the listed variable as independent variable, and trunk/limb fat mass ratio as the dependant variable

^b^For the multivariable model, explanatory variables were entered in the multivariable model if they showed an association (indicated by *p* ≤ 0.10) with and trunk/limb fat mass ratio in the univariable model, and kept into the final model if they were independently associated with and trunk/limb fat mass ratio or if they modified the point estimate for other predictors by more than 10%

For all coefficients showed in this table, the null value (showing no association) is 0

Table 2 also presents the results of the multivariable linear regression model. Predictors that were independently associated with a decrease in FMR were female sex and higher viral loads. Drugs that remained associated with increases in FMR were zidovudine, stavudine and raltegravir. Note that beta coefficients for increases in FMR are given for 10 years of exposure to the drugs. They can be interpreted as the predicted increased in FMR by this model that would be expected if a patient is exposed to the drug for 10 years.

### Continuous FMR analysis- comorbidities

Table 3 shows the mean expected variation in markers of metabolic dysfunction associated with each 1 unit increase in FMR. Average predicted increase in triglyceride levels was 0.39 mmol/l [95%CI 0.19–0.59], and in fasting blood glucose was 0.29 mmol/l [0.11–0.49]. There were not association between variations in FMR and LDL or HDL levels.

**Table 3:** Predicted variation in cholesterol and fasting blood glucose lever per 1 unit increase in trunk/limb fat ratio

	Coefficient [95% CI]	p-value
Triglyceride	0.39 [0.19;0.59]	< 0.001
LDL	0.07 [− 0.07;0.21]	0.33
HDL	−0.05 [− 0.10;0.01]	0.076
Fasting Blood Glucose	0.29 [0.11;0.49]	0.002

## Discussion

In this study, we showed that prevalence of LD remains high (47%) in our population. We found an association between LD (defined as FMR ≥ 1.5) and increasing age, male sex, Caucasian ethnicity and duration of HIV infection. When analyzing the association between cumulative exposure to ARV and FMR, we found that years of exposure to zidovudine, stavudine and raltegravir were independently associated to higher values of FMR, indicating more LD. We found no association with exposure to abacavir, tenofovir, lopinavir, darunavir, atazanavir, and ritonavir. Increasing FMR was also associated to an increase in blood triglycerides and fasting blood glucose, as well as with use of statins, fibrates and oral anti-diabetic drugs.

The prevalence of LD found in our study is in keeping with previous reports by Miller et al. from Australia (53%), and lower than reported in the Belgrade cohort study (69%) [8, 9], or than a study by Carr et al. where 83% of recipients of protease inhibitors developed LD [20]. Varying definitions for LD may contribute to varying estimates. Nonetheless, it remains an important and prevalent problem for people living with HIV. Our study showed that high viral load was associated to decreasing FMR (indicating less lipodystrophy). Miller et al. reported similar significant relation of viral load (< 500 log/ml) to LD. (*p* < 0.001) [9] and progressive probability of fat accumulation with lower peak viral load was also explained by Lichtenstein et al. [16] This finding likely reflects increased antiretroviral drug exposure.

The association of LD with zidovudine and stavudine has been widely reported before and was confirmed in a systematic review of randomized controlled trials [21]. However, the association found in our data between LD and raltegravir use is surprising. Indeed, Domingo et al. have shown improvement of limb fat and trunk/limb fat ratio when switching from stavudine to raltegravir containing regimens [7]. Martin et al. showed similar increases in limb fat when comparing regimens of 2NRTIs + lopinavir/r to to raltegravir+ lopinavir/r [17]. Similar findings are reported by McComsey et al., who found no difference in lean mass and regional fat when they compared atazanavir/r, darunavir/r and raltegravir on patients taking tenofovir/emtricitabine based regimen [22]. In a cohort study by Lake et al., 61 women living with HIV taking PIs and NNRTIs for 24 weeks were switched to raltegravir, and reported improvement of visceral adipose tissue in the abdomen and body image in immediate switch period, but not in delayed switch [23]. In view of the absence of LD associated to raltegravir in the literature, and due to the cross sectional nature of our study, it is possible that the association found between raltegravir and LD in our data is due to reverse causality: raltegravir may have been prescribed to very experienced patients with greater baseline levels of LD.

Strengths of our study include taking into account the duration of ARV exposure, and sufficient number of patients exposed to raltegravir. Furthermore, all patients underwent the same, objective testing at a unique clinical site.

Weaknesses of this study include its cross-sectional design, and multiple drug exposures, so that it is difficult to know if FMR was due to current, concurrent or past medication. As evidenced by our post-hoc power calculations, we lacked power to study all antiretroviral drugs, and, most importantly, to study drug combinations and interactions. Another limitation is that DEXA measures total trunk fat and does not allow to distinguish between visceral fat deposition (seen in LD) and subcutaneous fat deposition (which would give false positive for LD). Furthermore, DEXA cannot differentiate between abdominal fat accumulation due to LD or as part of the metabolic syndrome. This could explain the association seen between higher FMR and lipid and glucose metabolism abnormalities. As patients were offered DEXA scans as part of routine clinical setting rather than as part of a study protocol, the study population may not be fully representative. Patients with more concerns over bone health and LD might have been more inclined to undergo the test. While this selection bias could impact our estimates of LD prevalence, it should not impact the associations between medication and LD. Finally, duration of exposure to current or past medication was treated in the same way: models were not adjusted for years of non-use for some medication before DEXA tests; this may not be a good representation of the metabolic toxicity caused by these drugs.

## Conclusion

In this cross-sectional study, cumulative exposure to zidovudine, stavudine, and raltegravir were independently associated to higher FMR values, indicating more LD. The association with raltegravir is not supported by other research and warrants additional investigations.

## Data Availability

The datasets generated and/or analyzed during the current study may be made available from the corresponding author on reasonable request. The permission to access and use the raw data was granted by the Comité d’éthique de la Recherche du Centre Hospitalier Universitaire de Montréal.

## Abbreviations

ART: Antiretroviral therapy
ARV: Antiretroviral
CHUM: Centre Hospitalier de l’Université de Montréal DEXA dual energy
FMR: Fat mass ratio
HDL: High density lipoprotein
HIV: Human immunodeficiency virus
IQR: Interquartile range r ritonavir
LA: Lipoatrophy
LD: Lipodystrophy
LDL: Low density lipoprotein
LH: Lipo-hypertrophy
NNRTI: Non-nucleoside reverse transcriptase inhibitor
PI: Protease inhibitor
X-ray: Absorptiometry
